# Supplementary figures and images for: Genomic characterization of 99 viruses from the bunyavirus families Nairoviridae, Peribunyaviridae, and Phenuiviridae, including 35 previously unsequenced viruses
Source: PLoS Pathog. 2021 Mar 1;17(3):e1009315. doi: 10.1371/journal.ppat.1009315 (PMC7951987; doi:10.1371/journal.ppat.1009315)

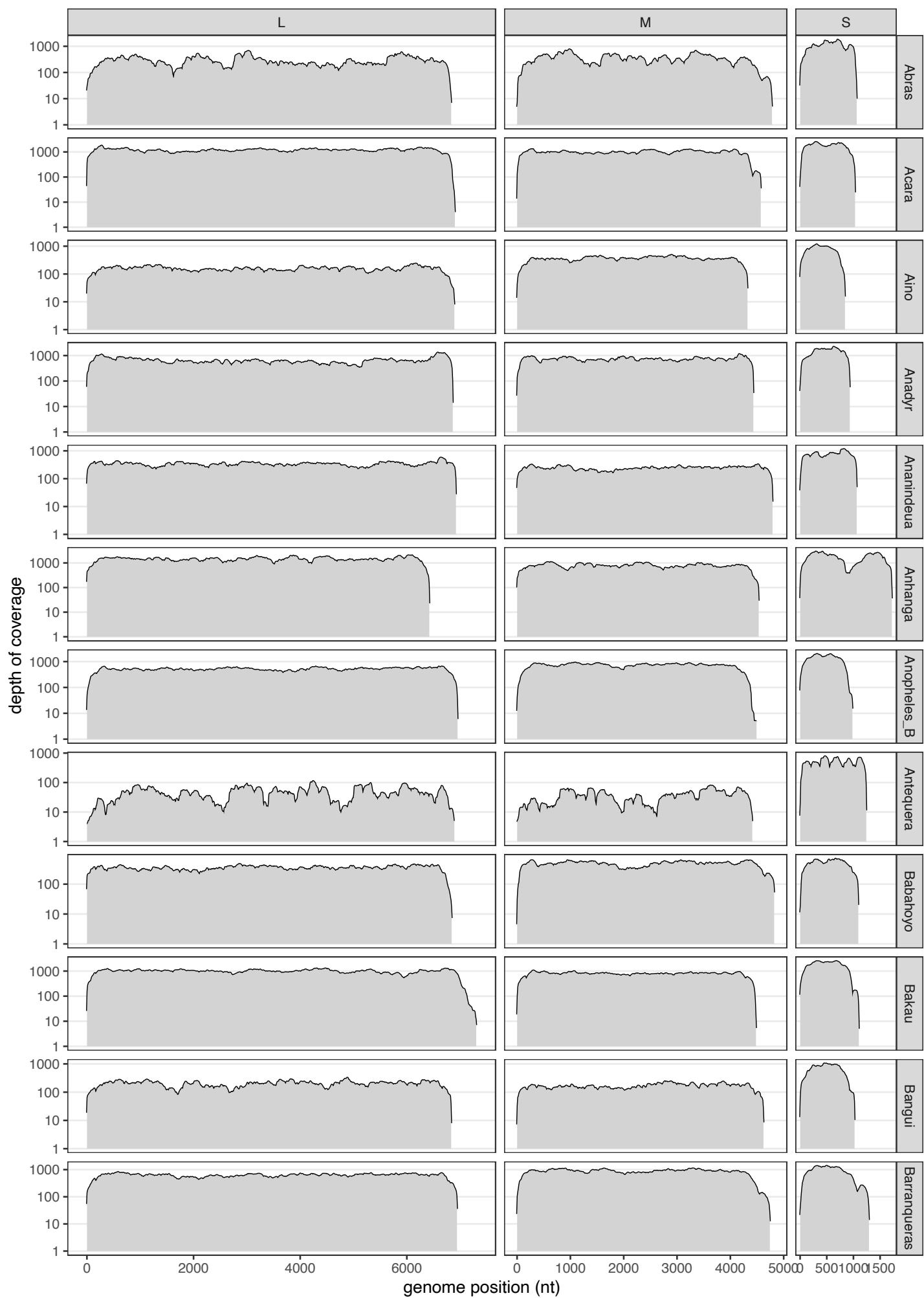

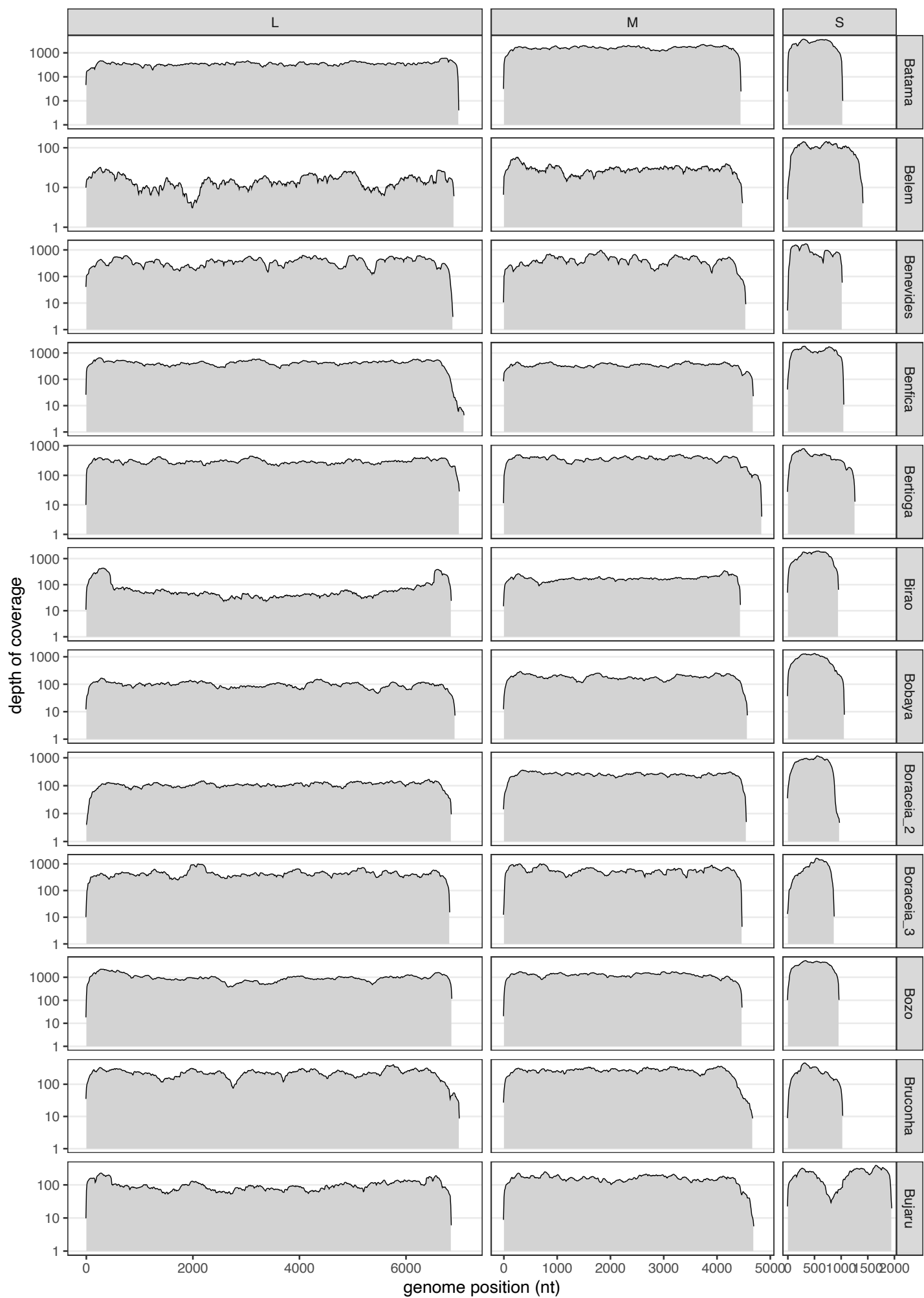

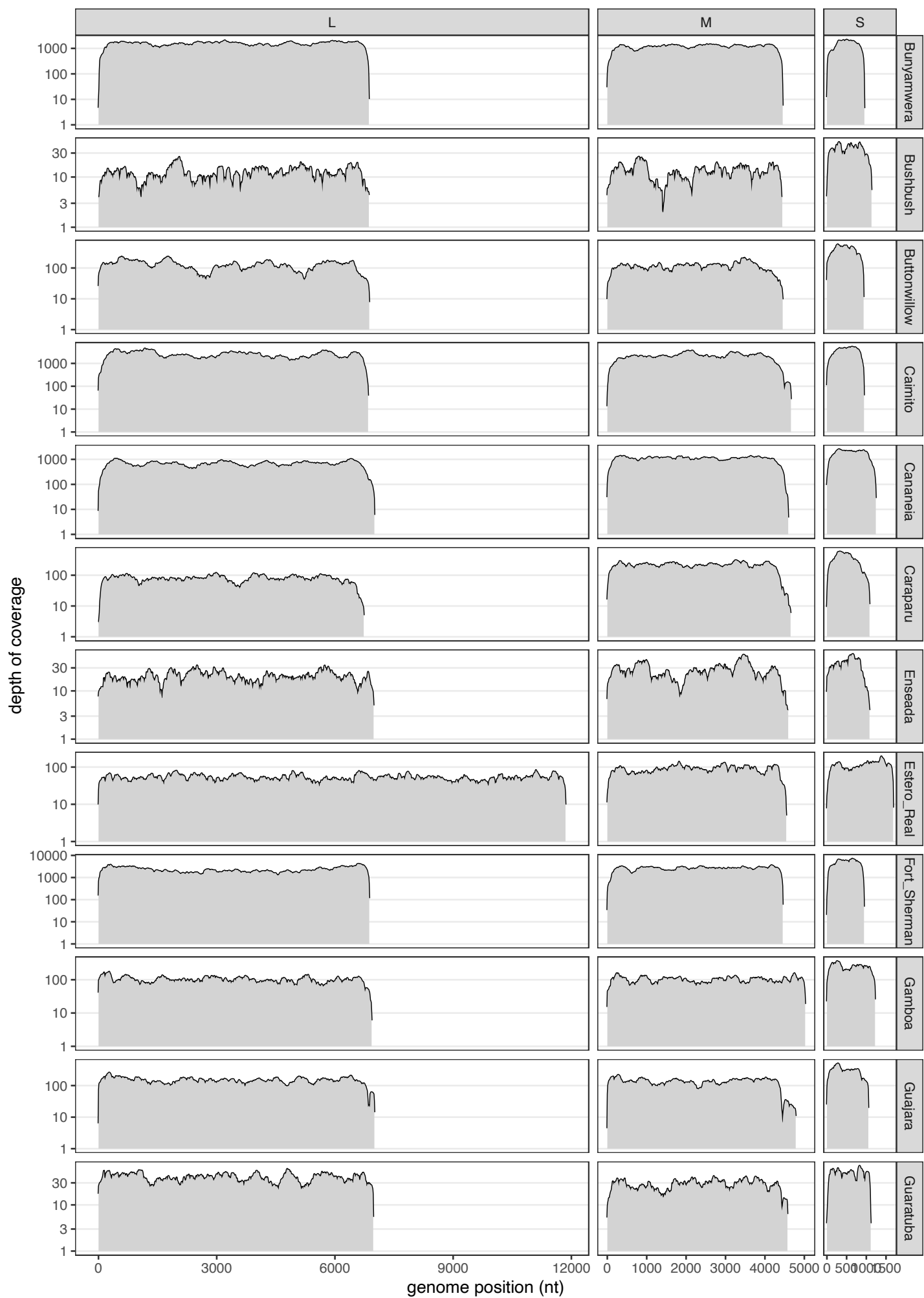

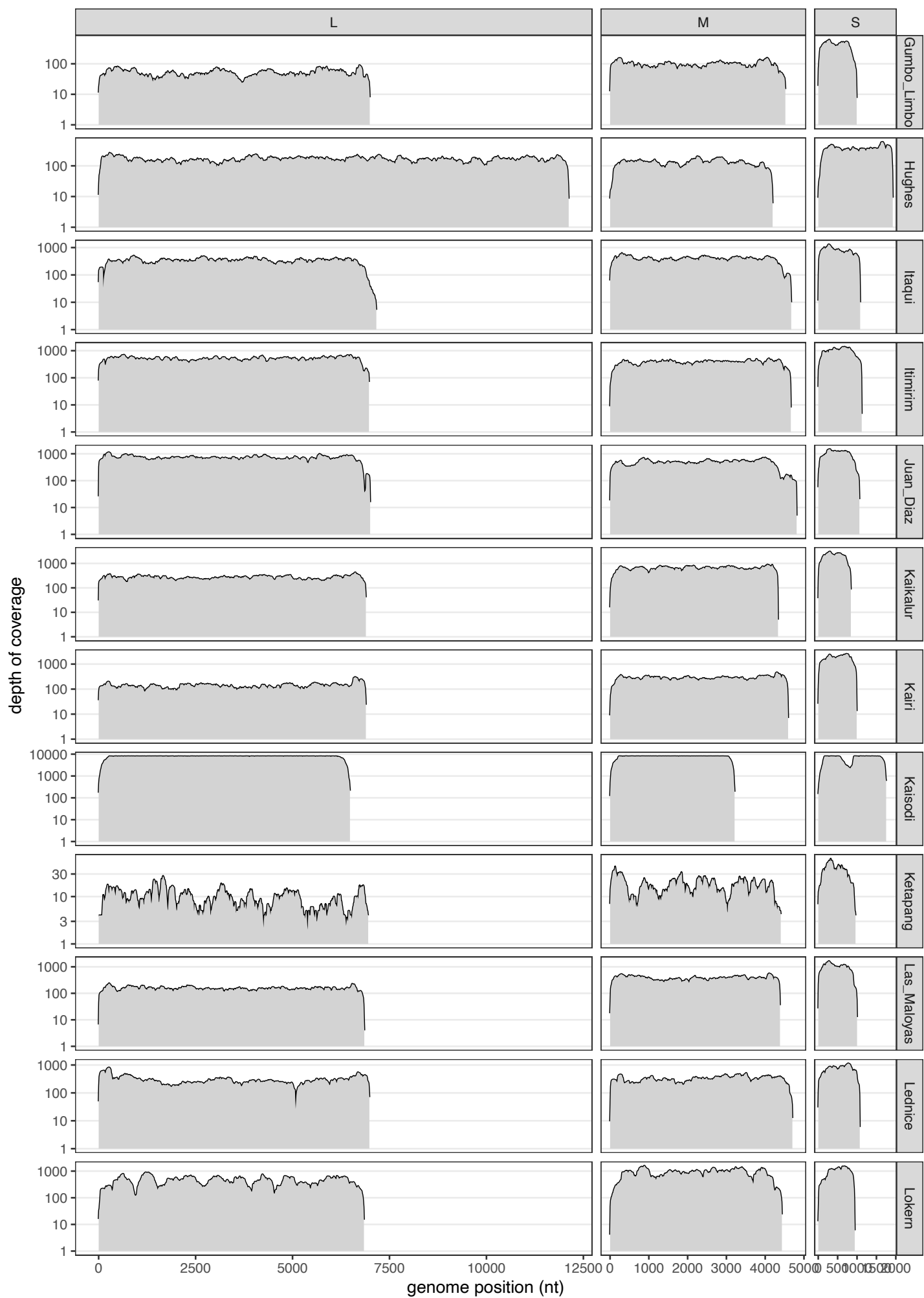

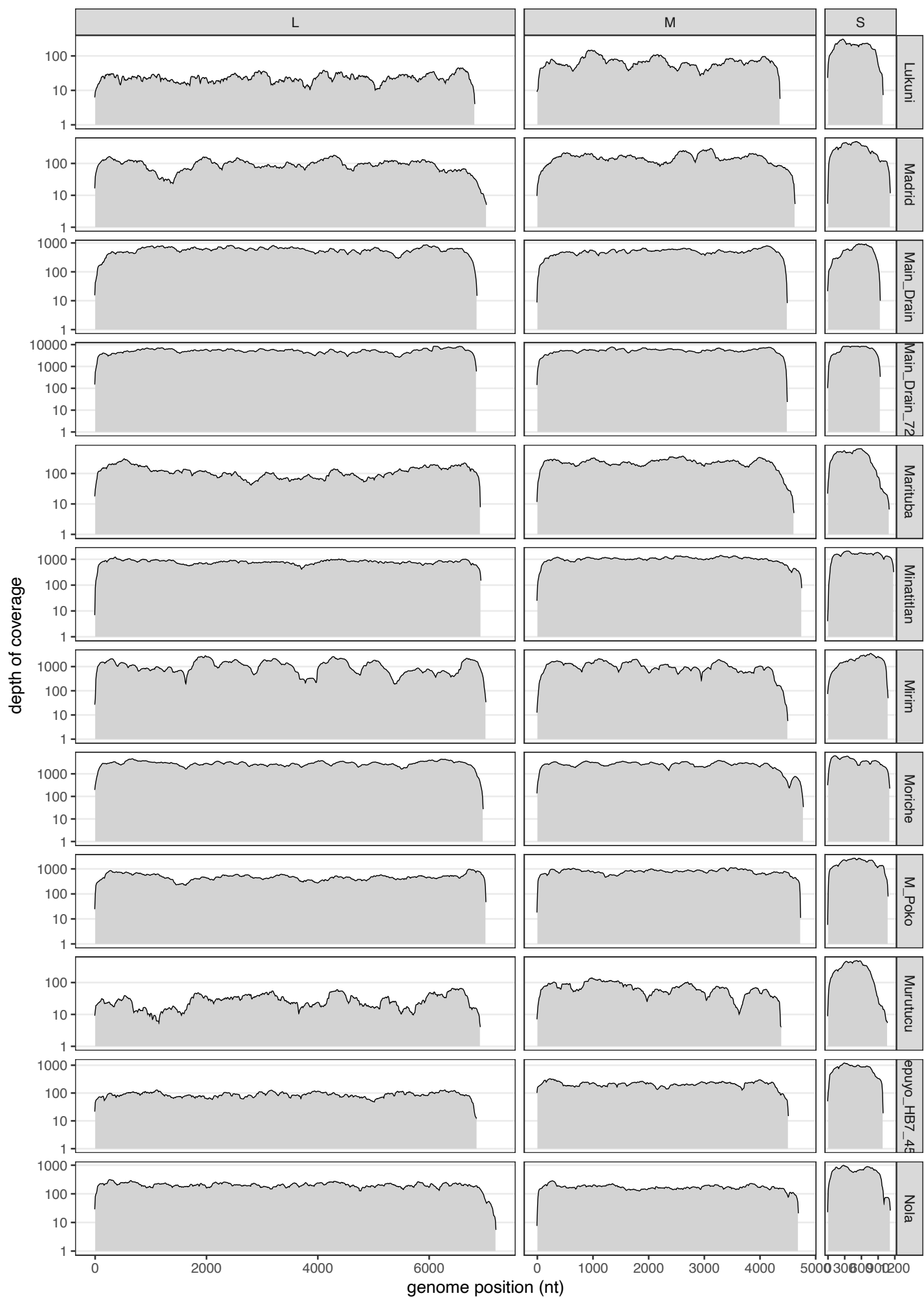

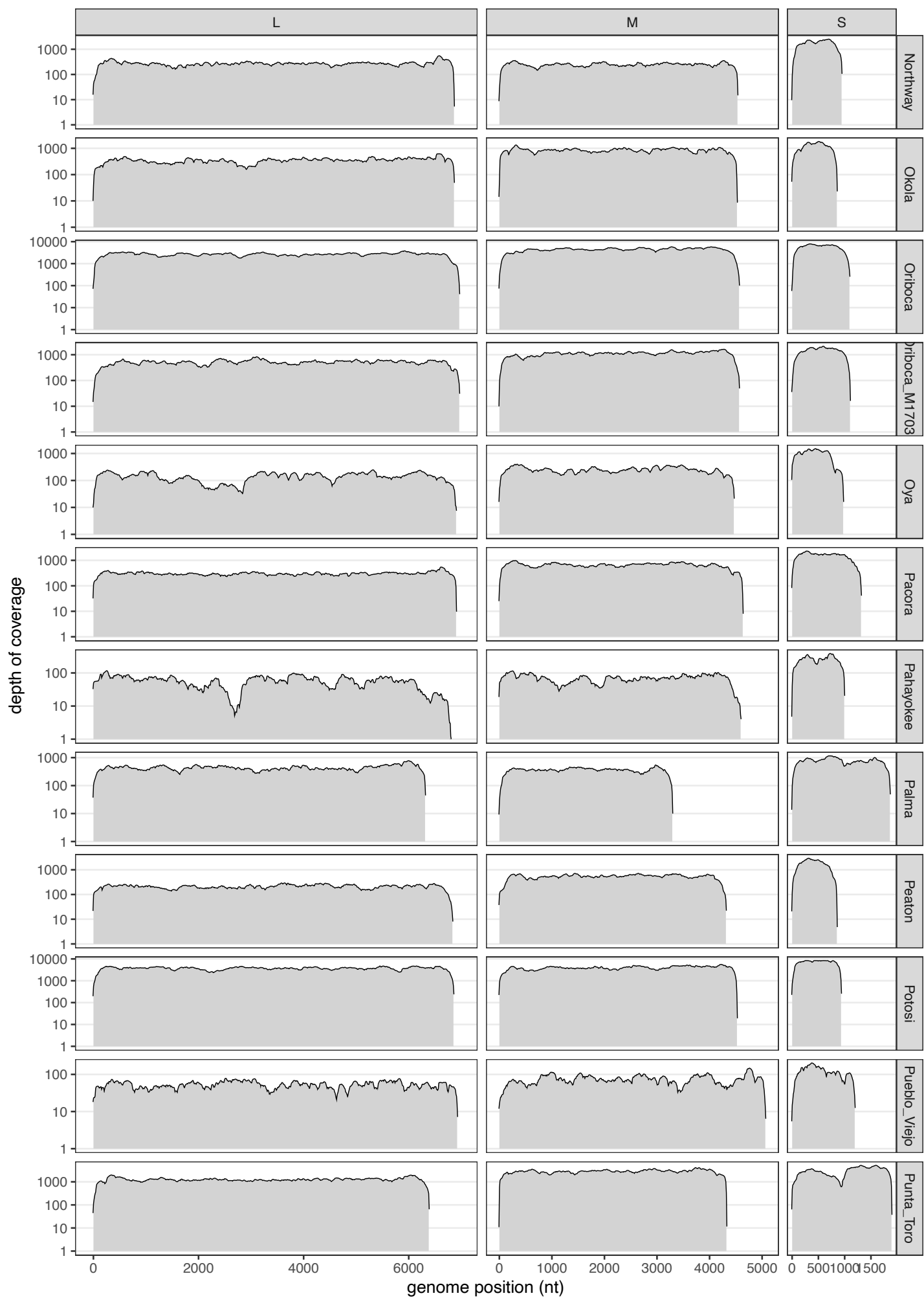

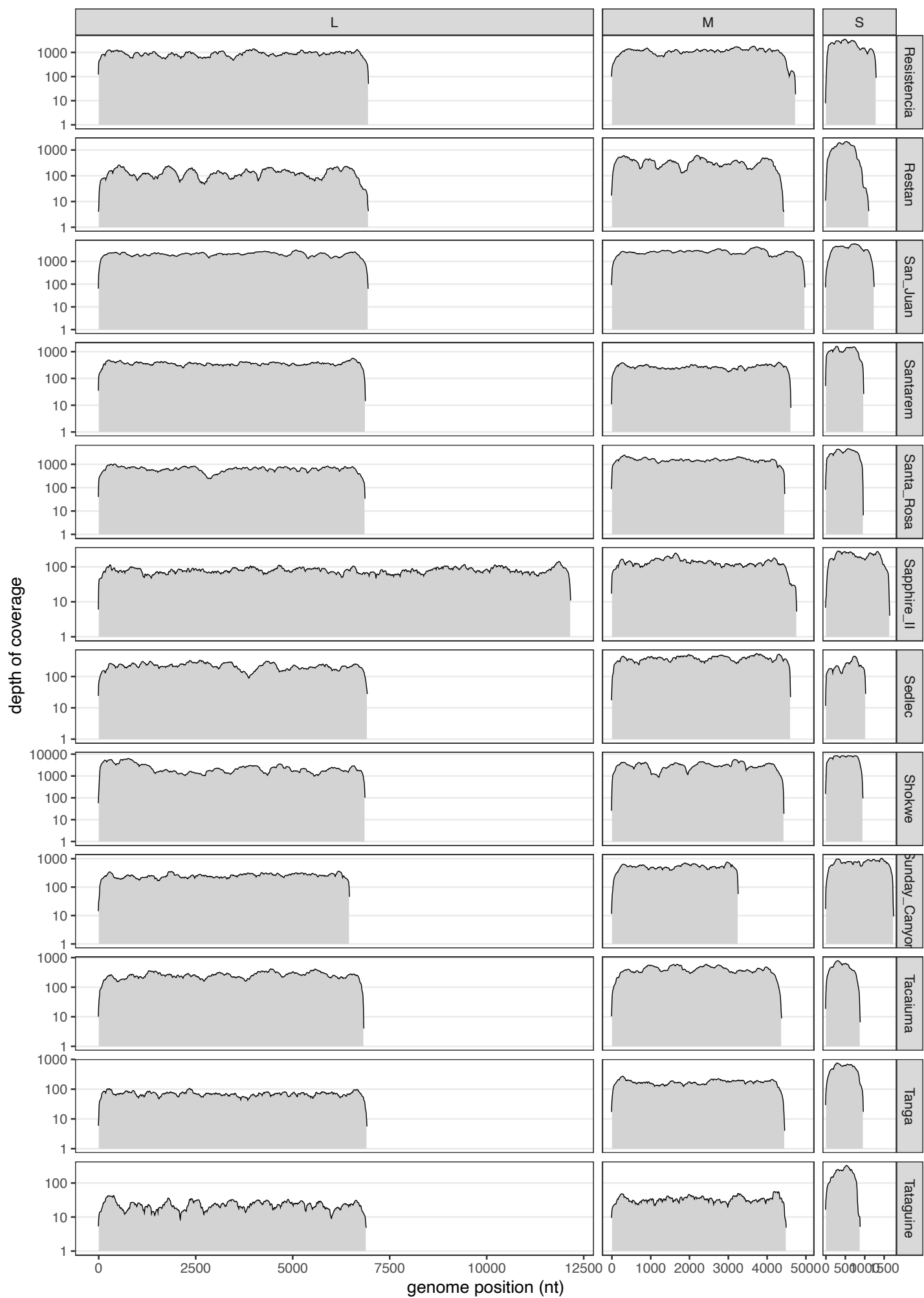

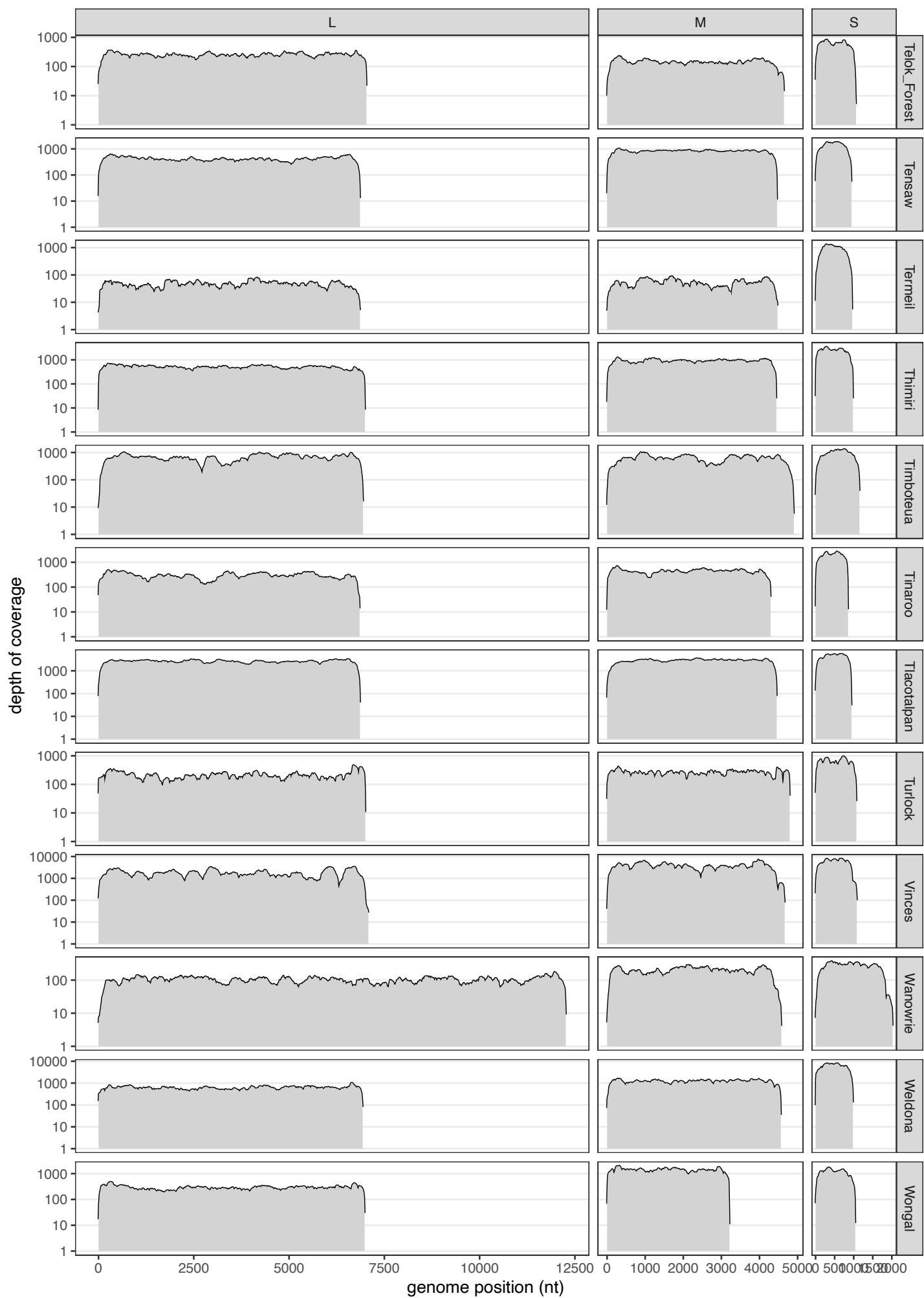

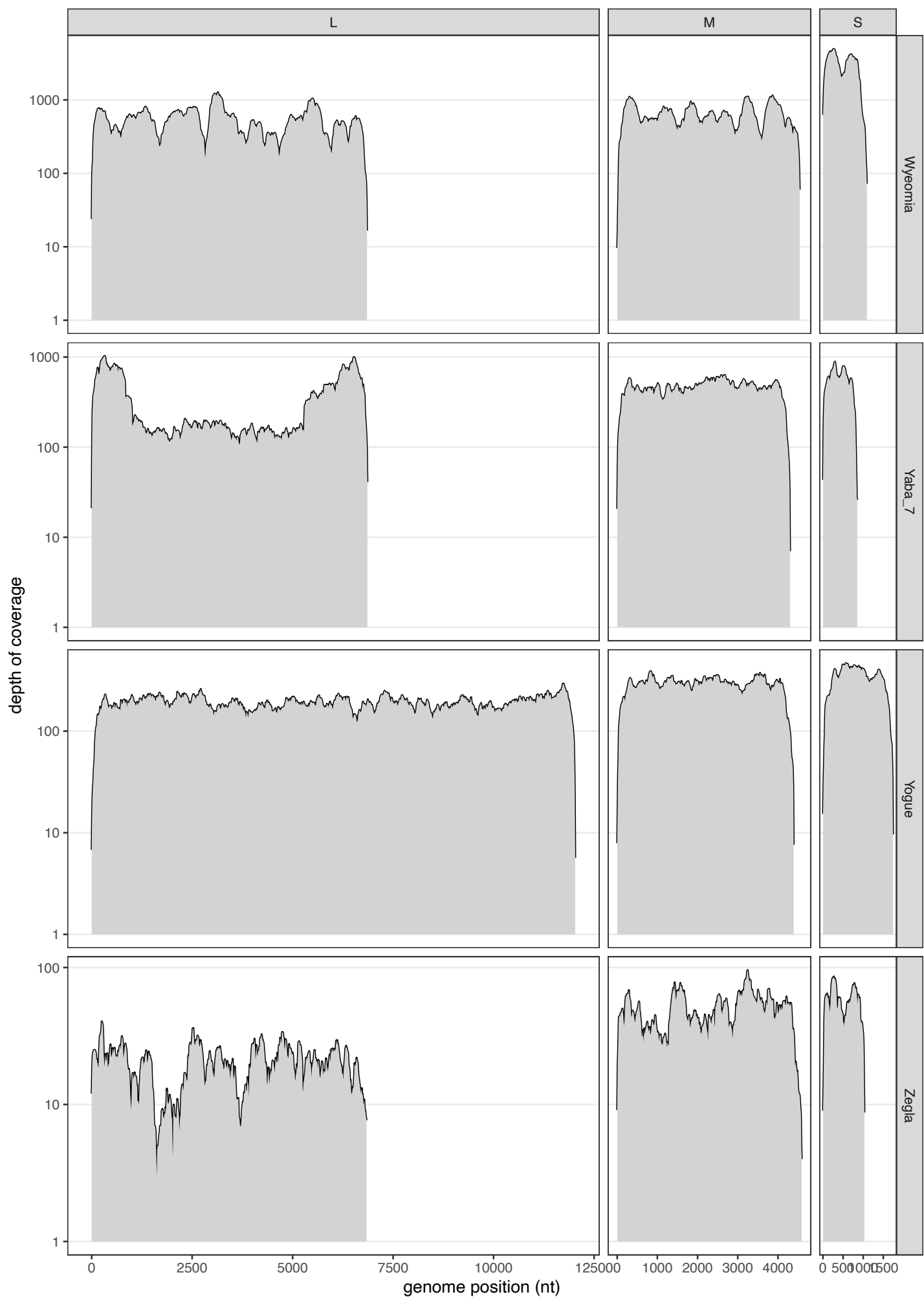

Supplement: S1 Fig — Coverage represents mean coverage for non-overlapping 10 nucleotide windows. (PDF) [file ppat.1009315.s001.pdf]

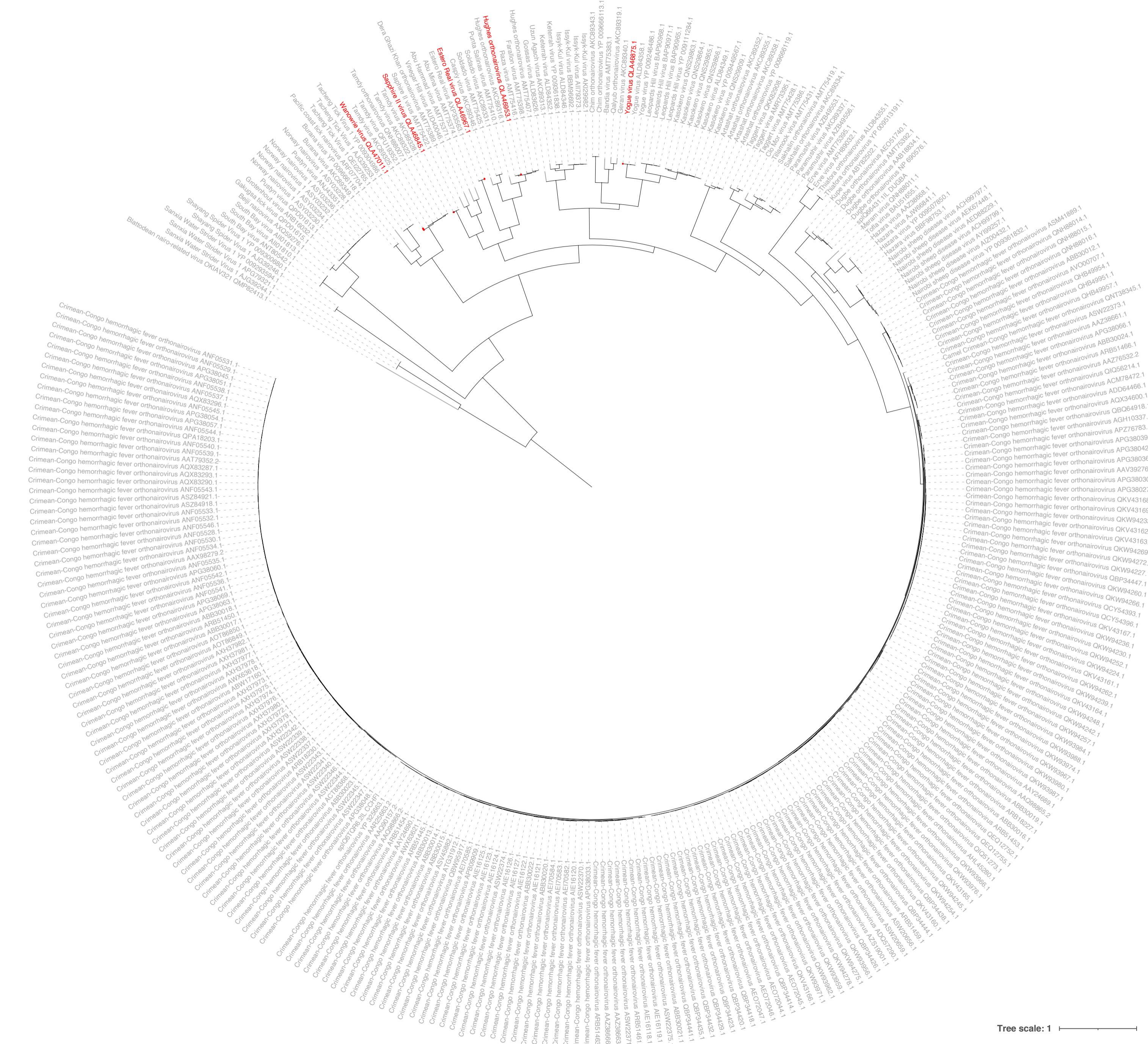

Tree scale: 1

Supplement: S4 Fig — All complete L protein sequences annotated under the Nairoviridae family in the NCBI Taxonomy database were downloaded and used to infer a maximum likelihood phylogeny. Sequences that we generated in the course of this study are indicated in red. Tree is midpoint rooted. Scale bar indicates substitutions per site. (PDF) [file ppat.1009315.s004.pdf]

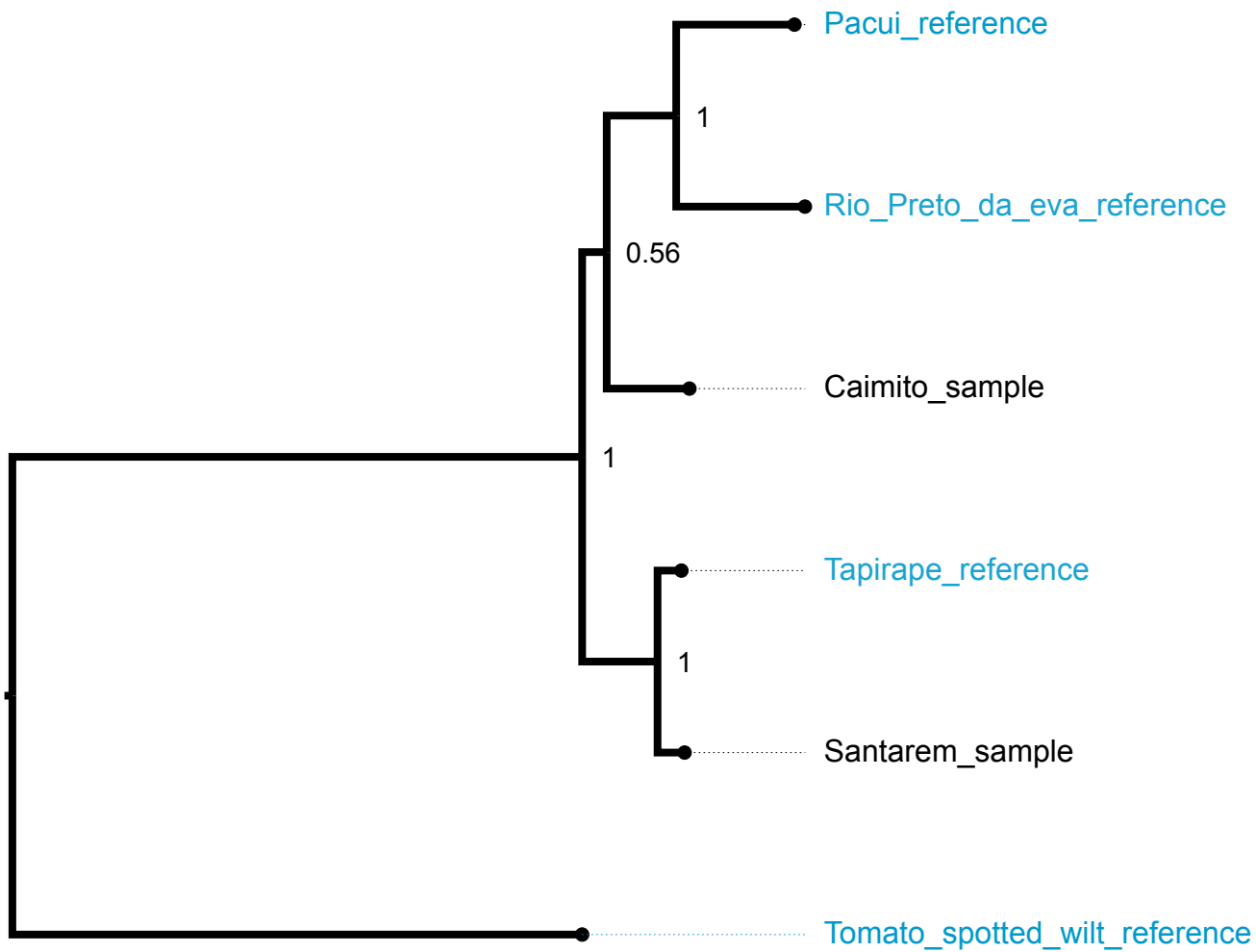

1.0

Supplement: S5 Fig — The virus sequences we generated in this study are shown in black. (PDF) [file ppat.1009315.s005.pdf]
