# Supplementary material for: Genomic characterization of 99 viruses from the bunyavirus families Nairoviridae, Peribunyaviridae, and Phenuiviridae, including 35 previously unsequenced viruses
Source: PLoS Pathog. 2021 Mar 1;17(3):e1009315. doi: 10.1371/journal.ppat.1009315 (PMC7951987; doi:10.1371/journal.ppat.1009315)
Supplement: Attachment — Submitted filename: Kapuscinski et al reviewers comments_2020_12_31.docx [file ppat.1009315.s008.docx]

Dear Dr. Kuhn and Reviewers

Thank you for your constructive feedback on this paper. We have addressed your concerns and questions as described in the detailed responses below. We believe that the paper is improved as a result, and thank you for considering this revised version for publication.

Mark Stenglein and Rebekah Kading, on behalf of the authors

**Part I - Summary**

Please use this section to discuss strengths/weaknesses of study, novelty/significance, general execution and scholarship.

**Reviewer #1:** This is an incredibly impressive manuscript where the authors sequenced and phylogenetically analyzed the complete ORFs of 99 bunyaviruses in the CDC’s archived collections. The viruses were collected over 66 years in multiple countries. I was particularly impressed with Figures 7 to 11 which showed all of ressortment events. I have never before seen ressortment data presented in this way before. All of my concerns are minor.

It was not immediately clear to me as to how many novel viruses were identified because they had previously been misidentified. Considering mentioning this in the abstract or towards the start of the discussion.

The contributions of each of these sequences to the public domain have been noted in Tables 1-3 as follows:

1 = Completely new genome sequence

2 = New strain sequence information

3 = Addition of missing genome segments or coding complete segments

4 = Sequence information already existed. In almost all of these cases, sequences were deposited by other authors during the course of this study.

In summary, we generated completely novel sequence for 35 viruses: 33 orthobunyaviruses, 1 phlebovirus, and 1 orthonairovirus. Additionally, we are contributing coding complete sequences of new viral strains for 26 viruses: 22 orthobunyaviruses, 3 phleboviruses, and 1 orthonairovirus. The sequence information generated for the remaining viruses adds missing genome segments or coding complete segments or the sequence information already existed. During the course of the study, some of the sequenced viruses were published by others. For the sake of completeness and contributing additional sequence data for broader use, we chose to still include our sequences and genetic analyses for these viruses. Given that viruses can change over passage through adaptation or contamination by co-infecting viruses, we felt that there was possible value in including these sequences that in theory, ought to be identical.

It could be worthwhile to mention the ICTV’s criteria for species demarcation (optional)

Species demarcation criteria for the bunyaviruses have historically been based on serological cross reactivity and increasingly on sequence similarity cutoffs. These criteria have been criticized for inconsistency across the order, restricted to only one segment, and being too all-encompassing by lumping distinct viruses together within the same species (PMID 29692303). Reassortment further complicates the issue. We contribute to a richer sequence set to make these decisions, but we believe that it’d be best to leave the actual demarcation to the relevant ICTV study groups, which have the appropriate official standing.

Page 35 explains that reassortment events usually arouse from M segment swaps. Others have reported which segment swaps occur most often. This prior work should be mentioned in the discussion

We have added citations to the relevant section of the discussion, which now reads:

“L and S segment trees were largely concordant, and reassortment was, in almost all cases, evident as pairs or groups of viruses that had similar L and S segments but different M segments. Previous studies have reported that reassortant bunyaviruses tend to combine the S and L segments from one parent with the M segment from the other parent, making this a seemingly common phenomenon (19,140–142), although this is not always the case (17). Linkage disequilibrium analysis of LACV isolates from field-collected mosquitoes showed evidence of frequent reassortment among strains in the field, and among all three genome segments (131). Among the viruses we analyzed, there were several examples of pairs of viruses that had nearly identical S segments but relatively different L and M segments, suggesting recent reassortment events that may have involved the joining of the S segment of one parental virus with the L and M segment of the second parent.”

Line 225 – I suggest deleting (N=2) and (N=6) from the text and instead say “1940 to 2005”.

We have revised as suggested.

Page 1 –Table 1 – so references are italicized, others are not

We have revised so all references are not italicized in Table 1.

Whenever “pairwise identity” is mentioned, it would be preferable if it were “pairwise nucleotide identity” to avoid confusion with “amino acid identity”

We agree with this suggestion and have made these changes – in all cases, pairwise is now followed by the relevant type of identity.

**Reviewer #2:** Kapuscinski and colleagues have sequenced several new viral genomes and supplemented sequence fragments to previously described arboviral species from a wide range of vertebrates and arthropods. The authors used a combination of Illumina- and Sanger-based sequencing approaches on a large repository of novel and ARC bunyaviruses. In some cases, plaque purifications were necessary to separate coinfections in vitro. Phylogenetic analyses using a Bayesian approach helped frame the sequencing contributions in an evolutionary and serological context. In particular, the co-phylogenies are well presented and bring attention to bunyavirus reassortment events.

Thank you very much for the positive feedback on this paper.

**Reviewer #3**: Kapuscinski and colleagues present a comprehensive study in which they determined coding complete sequences of 99 cell cultured Bunyavirus isolates. 35 of these coding complete sequences may represent new Bunyavirales species. For sure an interesting and important study, but mainly descriptive with a lot of data to comprehend. Data which is not always presented in a clear way, Apart from that, there is actually almost no comment to give on this manuscript. Interesting findings, methods are clearly defined and appropriate. Results and Discussion are substantial and sometimes difficult to follow.

Thank you very much for the positive feedback on this paper. We understand the Results and Discussion are information-dense. Revisions to improve the clarity and flow of the manuscript are described below.

**Part II – Major Issues: Key Experiments Required for Acceptance**

Please use this section to detail the key new experiments or modifications of existing experiments that should be absolutely required to validate study conclusions.

Generally, there should be no more than 3 such required experiments or major modifications for a "Major Revision" recommendation. If more than 3 experiments are necessary to validate the study conclusions, then you are encouraged to recommend "Reject".

**Reviewer #1:** All of my comments are noted above

Thank you.

**Reviewer #2:** Although there is a notable amount of novel genomic sequences reported in the manuscript, the authors may be unintentionally "overselling" their results in two main ways:

First, the number of “real” new viruses provided in this manuscript is 35 (counting “1” in Tables 1-3). This number is not mentioned anywhere in the text. If we count the 14 number “3”s in the same tables, it still is not even close to the consistently reported “99 viruses” that the authors present. The other numbers and the specific contributions of the authors should be elaborated in the text and tables. For example, in the case of “2”, does this equate to new metadata on the sample, or did the authors sequence a new isolate of an already catalogued virus while also adding some additional information? Looking at Bunyamwera virus from 2006, the complete genome sequence was deposited two years ago by different authors (MH484288-90), so I am not sure what was contributed from a genomic standpoint in this study. But then for Enseada virus, the strain name differs than the existing entries in GenBank and this seems like a new addition to Enseada virus genomic diversity.

It was not our intention to oversell this work. We included Fig 1E and the categorization scheme (1->4) in Tables 1-3 specifically to be clear about the newness of these sequences.

When we first undertook this effort in 2017, we selected viruses and strains for which genome sequence was partially or totally lacking. While we were in the process of conducting the study, other research groups sequenced 26 of the same viruses and strains (#4 in the tables). For that reason, the overall novelty of these sequences was a bit lower than we’d originally intended, but we believe the genome sequence data provided in this study still represent a significant contribution.

It’s worth noting that there are numerous examples of viruses that have been repeatedly sequenced. This includes numerous bunyaviruses that have been repeatedly sequenced (see new supplemental figures, described below): there are >200 full-length CCHFV sequences alone. There are also now something like 200,000 SARS-CoV-2 sequences. So there can be value in sequencing the same virus repeatedly. One could argue that sequencing that same virus *isolate* repeatedly is not useful, but virus isolates are not static: we identified evidence of co-infecting viruses that were not been by other authors who sequenced the same isolates/strains from a different source (e.g. for Abras virus), and viruses can evolve over passage.

We have also utilized these data to look for evidence of reassortment, and some redundancy in GenBank hopefully will enhance confidence in sequence validity for these strains as the result of independent sequencing efforts.

Regarding the strain of Bunyamawera virus referenced by the reviewer -- those are in fact sequences from our study: first author Kapuscinski was married after those sequences were deposited; her maiden name is Layton.

But we take the point that readers could misinterpret the overall novelty of our study. To further clarify, we’ve made the following changes:

We changed the title to:

*Genomic Characterization of 99 viruses from the Bunyaviral Families Nairoviridae, Peribunyaviridae, and Phenuiviridae, including 35 previously unsequenced viruses*

We added the following text as the 2^nd^ paragraph of the results, which defines how we classify “previously unsequenced” viruses based on similarity to existing database sequences:

“At the time of submission, 203 of the genome segment sequences we generated shared less than 97% pairwise nucleotide identity with existing sequences in the NCBI nucleotide database, as measured by blastn (**Figure 1E**). For 35 of the viruses, all 3 segments shared <85% nucleotide identity with existing sequences by blastn alignment. These 35 viruses represent previously unsequenced viruses (category 1 viruses in **Tables 1-3**; **Figure 1E**). For 37 of the viruses, different strains of the same viruses had been previously sequenced or existing sequences were not complete (e.g., only one segment had been sequenced; categories 2 and 3 in **Tables 1-3).** For 26 of the viruses, coding complete genomes already existed (category 4 in **Tables 1-3**). In almost all of these 26 cases, sequences had been deposited by other groups during the course of our study.”

We have also modified the concluding paragraph of the Conclusion paragraph as follows:

[old]: “In conclusion, this study contributed 99 genome sequences, many of which are new, to the public domain.”

[new]: “This study contributed 35 novel bunyavirus genome sequences to the public domain.”

We’ve also added a new phylogenies with all available bunyavirus sequences, as described in the response below.

No additional metadata were added to the ARC beyond the genome sequences.

For “4” why are these included in the “99” if they have already been fully characterized elsewhere? The scientific merit and relevance of including resequenced strains with already existing full genomes is not made clear here. This could be important is if the authors are using a primary sample or earlier cell passage than the current sequenced isolate. This would provide a more “authentic” genomic sequence, which could have evolutionary and possibly medical countermeasure implications. The authors should also consider directly linking new and existing viral isolates to the ARC.

Please see our response to the comment immediately above. We have also added passage history information to Tables 1-3 per this reviewer’s request. Viruses can adapt over passage and indeed, as we describe in the text, some of the viruses we sequenced contained co-infecting viruses, as an example of how different lots of what is ostensibly the same virus can change. Another recent example involves SARS-CoV-2, for instance strain USA/WA1, which adapts substantially during passage in culture. There are multiple Genbank entries for this virus corresponding to different passages.

Second, the estimated phylogenetic trees were complemented with publicly available sequences only from RefSeq, which is also “oversells" the true extent of the author’s contributions. This approach makes the author’s “new” black entries look overwhelmingly high compared with the blue entries (the ones previously sequenced). The authors should change the conclusions to better reflect the novel results of their presented work. The trees should include all available complete ORF sequences in GenBank/SRA to highlight existing bunyavirus diversity. This will provide a greater framework for the author’s contributions.

The reason we only included only RefSeq sequences is because including all available sequences creates trees with so many tips that the tip labels are far too small to read. For that reason we included the RefSeq sequences as representative “phylogenetic landmarks” as we say in the paper.

Regardless, creating trees with all available sequences is a good suggestion as a complementary way to contextualize the sequences we generated. We’ve added new trees as supplemental figures 2-4 that include all sequences classified under the relevant genera or families in the NCBI Taxonomy database. Because you can’t read the tip labels, we simply used red color to denote ‘our’ sequences. This depiction has the advantage of highlighting the areas of the tree where we contributed the most new sequences, in other words, where we have most filled in sequenced space. These trees also highlight the extent to which some bunyaviruses have been repeatedly sequenced and re-sequenced (i.e. large clades with essentially 0 length branches).

We added relevant text in the materials and methods describing how we collected sequences to make these trees and the following text to the first results paragraph in the Phylogenetic Analyses subsection.

“We also created trees containing all available sequences annotated under the relevant taxa in the NCBI taxonomy database (**Supplemental Figures 2-4**).”

Furthermore, there are numerous instances where credit was not given to the appropriate publication, particularly if the virus was completely or partially sequenced previously. While acknowledging the original discovery of a particular virus or serogroup is important, sequencing efforts have occurred globally over the last decade and should be equally acknowledged for their respective contributions.

Our intention for this section of the Tables was to provide the original publication that described the virus, not papers that have reported sequence. While we agree with the reviewer that sequencing efforts are important to recognize, it would be a continuously moving target to track down all of this information when readers can simply search GenBank to obtain the current information for each virus. For clarification we have changed the heading for the tables to read “Original Virus Description” instead of “Original Reference”. We have also double-checked these references to make sure those provided here match the records in ArboCat and filled in gaps when present.

Overall, I do think there are some important characterizations and new information that advance the field. However, the author’s claims need to be reassessed and clarified in several places.

Thank you, we hope we have addressed these concerns

**Reviewer #3**: I do not have any major issues.

Thank you.

**Part III – Minor Issues: Editorial and Data Presentation Modifications**

Please use this section for editorial suggestions as well as relatively minor modifications of existing data that would enhance clarity.

**Reviewer #1**: All of my comments are noted above

Thank you.

**Reviewer #2:** Other minor comments:

Line 100: “Viruses represented a subset of bunyaviruses catalogued within the ARC (22) (Tables 1-3).”

As mentioned above, it would be helpful to provide links to the ARC in the main tables for each virus listed. In the cases where new information was added, specific contributions should be elaborated.

The ARC inventory is searchable only internally to CDC employees through an Access database, however the information known about these viruses (prototype isolates) can be externally accessed through the Arbovirus Catalog (ArboCat) [https://www.cdc.gov/arbocat/](https://wwwn.cdc.gov/arbocat/). The viruses contained in the ARC collection can be found at [https://www.cdc.gov/ncezid/dvbd/specimensub/arc/index.html](https://nam01.safelinks.protection.outlook.com/?url=https%3A%2F%2Fwww.cdc.gov%2Fncezid%2Fdvbd%2Fspecimensub%2Farc%2Findex.html&data=04%7C01%7CRebekah.Kading%40colostate.edu%7C52cdfd8a9b6c4dd7fc7108d884d4da27%7Cafb58802ff7a4bb1ab21367ff2ecfc8b%7C0%7C0%7C637405397036368773%7CUnknown%7CTWFpbGZsb3d8eyJWIjoiMC4wLjAwMDAiLCJQIjoiV2luMzIiLCJBTiI6Ik1haWwiLCJXVCI6Mn0%3D%7C1000&sdata=fViQtNJyV8TufA%2FFjwVA%2BH2fIqtUzIvMsDLn6UiJkCY%3D&reserved=0). We have added the website links to the manuscript, as well as mentioned that the taxonomy may differ in the ARC and ArboCat as the taxonomy is updated by ICTV. (p.8) The tables already contain a column that codes the contribution of each sequence to the public domain:

1 = Completely new genome sequence

2 = New strain sequence information

3 = Addition of missing genome segments or coding complete segments

4 = Sequence information already existed. In almost all of these cases, sequences were deposited by other authors during the course of this study.

We’ve also added a new second paragraph to the results section, as described above, which enumerates the numbers of viruses that fall into these different categories and the criteria we used for defining a ‘new’ virus sequence.

Line 109: “The KAPA RNA HyperPrep Kit was used to prepare sequencing libraries from total RNA according to the manufacturer’s protocol using half-scale reactions (Kapa Biosystems).”

What type of adapters were used? What measures were taken to limit adapter bleedthrough and ensure that reassortments were not as a result of laboratory contamination? In the Discussion, the author’s say that contamination has been ruled out, but they do not provide specific details and safeguards to ensure this.

We’ve added a description of the adapters we used to the relevant sentence in the materials and methods: “… using half-scale reactions and the Kapa Dual-indexed Adapter Kit.”

We have experienced index hopping / barcode bleedthrough and are aware of the problems it can produce in downstream analyses. Here, though, we are reporting consensus sequences supported by high coverage. In situations where there were co-infecting viruses with relatively low coverage, which you could argue could result from misassigned reads, there were two pieces of evidence that strongly supported this *not* being the case: (1) we used PCR to confirm all co-infecting viruses (described in the paper); (2) in all cases the sequences of co-infecting viruses were unique relative to all the other viruses we sequenced: if they were the result of index hopping, they would have the same sequence as another one of the high-coverage viruses we sequenced.

Line 124: “Viral contigs were further assembled using Geneious v11.0.2 as necessary (32).”

What settings were used in Geneious? Did you have a minimum depth cutoff? How were consensus sequences determined in low coverage areas? You mention the 4x cutoff at the ends, is that used throughout? If so, how were disagreements handled e.g. four total reads mapping two Adenines and two Guanines at the same position?

It was incorrect to say that we performed assembly in Geneious: we only validated sequences in Geneious. We’ve corrected the relevant section of the M&M as follows:

[old]: “Viral contigs were further assembled using Geneious v11.0.2 as necessary (32).”

[new]: “Viral contigs were validated by inspecting alignment of mapped reads to draft assemblies in Geneious v11.0.2 (32).”

And

[old]: “Bases at the ends of genomes with less than 4x coverage were trimmed.”

[new]: “Bases at the ends of genomes with less than 4x coverage of the same base were trimmed.”

Line 132: “…and alignments were independently validated by two people.”

I do not understand the significance of a two person validation.

We had two people inspect assemblies in Geneious as described above to validate them.

Line 172: “Poorly aligned, divergent positions characterized by excessive gaps in the alignment were removed using the Gblocks server under less strict conditions (37).”

First, I do not see any link or access point to alignments, which are crucial to the later phylogenetic inferences. The authors stated that all data should be open and should follow this. Second, I don’t think removing diverging positions or gaps is correct. Each site plays a role in reconstructing phylogenetic relationships and they should not be removed, especially in comparisons among distinct viral species.

The subject of how to best create phylogenetic trees is controversial. None of the conclusions we make in the paper are particularly sensitive to the small differences in tree topology that could result from using different phylogenetic methods. Analysis of reassortment could be incorrect if it were based solely on tree topology. For this reason we did not base our description of reassortment only on tree topology: we also used differential pairwise identities between segment pairs, and limited our descriptions to clearly well supported examples, as described in the paper. And in the tanglegrams we present, we collapsed branches to polytomies whenever support values were <95% to further guard against this possibility.

Line 216: “Single virus stocks were cultured from plaques that were sequence-confirmed to have only one of the infecting genotypes present.”

How were they sequence confirmed?

Plaques were screened using unique primers designed to each segment of both viruses, and amplicons were sequenced. Sanger sequence confirmation was added for clarification in line 225.

Line 235: “Some viruses were passaged through grivet (Chlorocebus aethiops) Vero or hamster (Mesocricetus auratus) BHK-21 cell cultures, others were passaged in suckling laboratory mice.”

Referring to an earlier comment, it would be nice to have passage histories available of the newly discovered virus stocks generated as part of this study.

The passage histories have been added to Tables 1-3 as described above.

Line 231/Figure 1E: “203 of the genome segment sequences we generated shared less than 97% pairwise nucleotide identity with existing sequences (Figure 1E).”

The authors discuss a lot of taxonomical implications in the manuscript. They may consider expanding this section by including a DEmARC analysis or other taxonomically related tool to differentiate new species and genera.

Please see the response above from another reviewer on this point. Analyses along these lines in a paper do not have authority outside of the auspices of the ICTV, and we feel that this would be best left for a Taxonomic Proposal to the relevant study groups.

Lines 280-287. I think this paragraph should be moved and merged into the Methods section. There is also some information here that isn’t explicitly stated in the actual Methods.

This paragraph has been moved to the Methods as suggested.

Line 285: “…which produced 748-fold mean coverage depth across virus genomes.”

The authors should include the coverage depth or possibly coverage plots for each virus somewhere in the manuscript or supplementary materials.

We’ve added coverage plots for all viruses and segments as a new supplemental figure.

Line 326: “This observation supports the hypothesis that this group of viruses resulted from an ancestral reassortment event involving the replacement of an S segment, as has been proposed for Brazoran virus (117).”

This is more of a discussion point than a result. I would move it to the Discussion or remove it.

This sentence has been moved to the Discussion as suggested.

Line 422: “In the Bakau serorgroup…”

Serogroup?

This has been corrected.

Line 437: “The Tinaroo and Akabane virus L and S segments were relatively closely related, but the M segments shared only 65% pairwise identity.”

I would add a percentage or some quantification to “closely related” to match the last part of the sentence.

The relevant portion of this sentence has been changed to read: “The Tinaroo and Akabane virus L and S segments were 87 and 95% identical…”

Line 461: “There was little evidence for reassortment among the orthonairoviruses in our analysis.”

Why did the authors opt-out of using the RDP4 package to test for reassortment? I am not disagreeing with the author’s approach, but RDP software has been used previously to confirm reassortments in bunyaviruses.

We did try to use RDP4 software (and have used it in the past to identify recombinants and reassortants) but this did not work well here, we believe because of the overall high level of diversity of sequences included in this study: clear examples of reassortant viruses, including previously published examples, were not identified using RDP4. So the approach we used proved to be an alternative that empirically worked better for these sequence sets.

Line 543: “The partial M sequence had a normalized coverage level of 2.4% relative to the full-length M sequence.”

Please clarify the meaning of this statement.

We changed this to read: “The partial M sequence had a coverage level of 2.4% relative to the full-length M sequence.” Normalized referred to the total dataset size, which in this case was the same, so the rations of normalized coverage is the same as that for coverage.

**Reviewer #3**: It is in the nature of a report of this type to focus on perceived shortcomings and incompleteness. My comments should be seen in the context of improving an already good manuscript.

Thank you!

* lines 100-117: I was surprized no additional depletion steps were included apart from DNA depletion after RNA extraction for suckling mouse brain preparations. The KAPA RNA HyperPrep kit was used for both sample types without rRNA or mRNA depletion steps, is that correct?

Yes: as we describe in the paper, and in the new supplemental figure showing coverage, this produced plenty of coverage to reconstruct these genomes. This is a bit of an aside, but in our experience, complicated and expensive depletion or enrichment steps are rarely actually necessary for viral metagenomics, unless you were doing something like trying to study low frequency variants or very low abundance viruses.

* I realise that Tables 1 to 3 are already extensive, but it would be good adding the corresponding GenBank accession numbers here as well. Also mark if segments are coding complete or full-lengths. It is actually confusing that most GenBank entries are marked with "complete sequence" but are apparently coding complete.

If available, a strain passage number before sequencing would be of interest as well... and overall coverage of segments.

The passage history and GenBank accession records have been added to Tables 1-3. We apologize for the confusion; the sentence “All sequences deposited in GenBank are coding-complete.” has been added to line 141 for clarification.

* GenBank entries also miss sampling date, species of origin, sampling location, ...

At the expense of going back and amending all of the GenBank records, we hope that adding the GenBank accession numbers to the Tables 1-3 will help readers associate all of these metadata with the isolates in question. We have also added the website addresses to the ARC and ArboCat for additional details on these viruses.

* For several of the isolates, 1 or more segments were verified with Sanger sequencing. What was the reason? Low coverage or incomplete assembly?

Sanger sequencing was performed to independently confirm that the sequences derived from the Illumina data did indeed come from the intended virus stock. For this confirmation, RNA was re-extracted from each virus. This process is a standard quality control check practiced at the CDC.

Lines 139-142 have been modified to read: “Independent Sanger sequence confirmation from re-extracted RNA was obtained from at least one genome segment for 71 of 99 viruses (197 segments). This validation was performed to ensure that the sequencing data were derived from the intended virus stocks, especially when working with such a large sample set.”

Did the authors attempted to determine the 5`and 3`panhandle structures and down/upstream regions? It is actually a shame that did was not done.

We agree, but unfortunately did not have the budget in this project to follow up on this.

* line 169-170: This makes no sense. Amino acid sequences are aligned with muscle, after that 'manually aligned afterwards using Seaview'...? Should this read 'manually corrected'? If so, why a manual correction and what was corrected? otherwise, why align 2 times?

Why are alignments converted back to nucleotides after aa alignments. How was this done and was the original nt sequence used or a generic codons? Why not use a better multiple alignment algorithm that can work better with divergent datasets and use the amino acid alignment as a guide for nucleotide alignment.

We appreciate these comments. This sentence should read “manually inspected”, as no further alignment adjustments were necessary after the muscle alignment. We chose to conduct an amino acid alignment due to the amount of divergence among the different virus isolates. Aligning to amino acid, rather than nt sequence, keeps the alignment in-frame and aligns the sequences conservatively. Seaview allows for the active conversion between amino acid and nt while maintaining the original nt sequence (not back converting to generic codons), we used this functionality to align to amino acid and transition back to nt. To clarify this point we included the word “original” on line 182 prior to the words “nucleotide sequences for phylogenetic analysis.” Regarding on the choice of alignment algorithm chosen, we believe Muscle was an appropriate alignment algorithm, The Phylogenetic Handbook (ed. Lemey, Salemi, and Vandamme) indicate on Table 3.2 that Muscle is an appropriate algorithm for the type and quantify of sequences aligned.

* For the Bayesian analysis performed, it is unnecessary to include an outgroup in this type of analysis. This can also introduce unnecessary disturbance and excessive gaps in the alignment.

We believe that it was appropriate to use an outgroup. Use of an outgroup was used to help resolve the polarity of the phylogenetic trees. We were very careful in choosing the outgroups used so as not to create excessive gaps and disturbances in the alignment, noting outgroups used for each genus can vary. Furthermore, the outgroup command in Mr. Bayes merely affects the display of the trees, rooting the trees to the outgroup and providing the polarity characteristics desired. To account for this concern we have included the following statement prior to delineating the outgroups on Line 177, “Outgroups were used to root and provide polarity characteristics to the phylogenetic trees. Outgroups were chosen so as to not provide excessive gaps and disturbances in the alignments.”

* line 187-216. Another strategy would be looking at variability in 5' and 3' panhandle structures.

Agreed.

* Define scale bar in phylogenetic tree figure legends.

Scale bar represents the number of substitutions per site (this text has been added to the relevant figure legends).

* Figure 5: which hantavirus was used as outgroup?

Apologies, the virus used was Hantaan virus. We have changed the legend of Figure 5 to clarify: “Outgroup: Hantaan virus (RefSeq: NC_005222, NC_005218, NC_005219).”

* The manuscript describes a lot of data. It is not always easy and clear to follow the results and discussion sections. Maybe consider the use subheadings in both sections.

A subheading for “Reassortment” was added to the Results, and subheadings were added throughout the Discussion.
